# Frailty Index in the Colonias on the US-Mexico Border: A Special Report

**DOI:** 10.3389/fmed.2021.650259

**Published:** 2021-08-18

**Authors:** Eron G. Manusov, Carolina Gomez De Ziegler, Vincent P. Diego, Gerardo Munoz-Monaco, Sarah Williams-Blangero

**Affiliations:** ^1^Department of Human Genetics, School of Medicine, University of Texas Rio Grande Valley, Edinburg, TX, United States; ^2^Knapp Family Medicine Residency Program, University of Texas Rio Grande Valley, Edinburg, TX, United States; ^3^South Texas Diabetes and Obesity Institute, University of Texas Rio Grande Valley, Edinburg, TX, United States

**Keywords:** Mexican American, quality of life, chronic illness, hispanic, US-Mexico border background

## Abstract

Frailty is the age-related decline in well-being. The Frailty index (FI) measures the accumulation of health deficits and reflects biopsychosocial and cultural determinants of well-being. Frailty is measured as a static phenotype or as a Frailty Index comprising a ratio of suffered health deficits and total deficits. We report a Frailty Index calculated from routinely measured clinical variables gathered from residents of two Colonias (neighborhoods) in South Texas. A Colonia is a predominantly Hispanic, economically distressed, unincorporated neighborhood. We analyzed retrospective data from 894 patients that live in two Colonias located on the Texas-Mexico border. We calculated the FI with seven physiological variables, PHQ-9 score, and the 11 domain-specific Duke Profile scores, for a total of 19 possible health deficits. FI against age separately in males (*n* = 272) and females (*n* = 622) was regressed. Females had a significantly higher starting frailty, and males had a significantly greater change rate with age. FI against age for Cameron Park Colonia and Indian Hills Colonia was regressed. We calculated a significantly higher starting FI in Indian Hills and a significantly greater change rate in Cameron Park residents. Frailty's contributors are complex, especially in neighborhoods of poverty, immigration, low education level, and high prevalence of chronic disease. We report baseline Frailty Index data from two Colonias in South Texas and the clinical and research implications.

## Introduction

Socioeconomic status, poverty, social determinants of health, and health disparities contribute to Frailty and affect healthcare planning and delivery. Frailty is associated with chronic disease, aging, and quality of life. We can define Frailty as a biological phenotype with three or more components (e.g., unintentional weight loss, self-reported exhaustion, weakness, slow walking speed), (1) or as a measure of health that considers the contribution and accumulation of multiple deficits (Frailty Index) that impact the quality of life and risk of death (2–4). The Frailty Index (FI) may incorporate social, physical, and psychological contributors, such as the use of multiple health services, hospital-acquired complications, worsening health, and loss of independence (4–9), as well as conditions such as cognitive impairment (5), cancer (6), dialysis (7), heart disease (8), and metabolic syndrome, (9, 10). The use of a Frailty Index can identify known and unknown contributors to health and well-being targeted in healthcare delivery and research across the lifespan. Multiple variables, such as age, chronic illness, health, and well-being, contribute to Frailty, and increased Frailty, is associated with higher mortality (11–24).

In studies involving over 1.5 million participants, various Frailty Indices are calculated based on as few as twenty and up to as many as one-hundred-thirty factors. Studies using multiple factors to calculate the Frailty Index are consistently associated with the risk regardless of the specific factors measured (25–27). Although often used to characterize health in the elderly, the FI also can be used to measure health at younger ages and the FI trajectory can be monitored as deficits accumulate (28). The Frailty Index's flexibility applied to a population can identify deficits unique to the region served.

There are a disproportionate number of patients with chronic illness and disease on the Texas- Mexico border region. Obesity (53.8%); hypertension (38.9%); diabetes (28.8%); and depression (21.8%) are epidemic in the area (29–31). One in three people in the South Texas Rio Grande Valley (a region located on the US-Mexico border) are uninsured or underinsured, and 40 percent of families in the region live below the poverty level. The area includes a large number of Colonias (unincorporated Hispanic neighborhoods that may lack basic services and that are characterized by nutritious-food deserts, greater exposure to infectious disease, limited transportation capacity, poor internet capacity, social vulnerability, and low social capital) (32). These Colonias are identified as “green” (low public health risk) and “yellow” (intermediate risk with inadequate city services, road paving, flood drainage, and waste disposal).

Demographic characteristics of this self-selected cross-sectional cohort and description of services provided are reported in earlier work completed by our research team (31). The purpose of our community case study is to describe a Frailty Index calculated from data routinely collected by primary care teams. We then use the Frailty Index to identify contributors to Frailty in predominantly Mexican American Communities residing on the Texas-Mexico border. The results can be used to design future research on the development of a clinical Frailty Index and identify interventions to reduce Frailty in vulnerable populations.

## Methods

The protocol was approved by the University of Texas Institutional Review Board. All patients signed a consent for care.

### Clinical Care

VIDAS *(Valley Interprofessional Dedicated Access and Service)* was funded through a grant to develop a Colonia Care Program. VIDAS's clinical arm serves to unite the region by building a consortium of professionals collaborating as a team to create a healthcare model for the community's most vulnerable members. The UniMóvil mobile clinic was created to address access to healthcare across the Colonias throughout the region.

The UniMóvil provides care in two Colonias, each with significant differences. Cameron Park was selected as the representative “green” Colonia because of a long history of an established indigent care model and the proximity to the Mexico border that facilitates ready access to affordable healthcare in Mexico. Indian Hills, a “yellow” Colonia, was chosen because of the location (distance from the border and poor healthcare access), low social capital, low socio- economic reserve, and poor transportation resources.

The team travels to the Colonias twice a month and provide routine primary care. As part of clinical care, we collect body mass index (BMI), systolic blood pressure, diastolic blood pressure, glycated hemoglobin (HbA1c), total cholesterol, LDL-cholesterol, HDL-cholesterol, triglycerides, glucose concentration, health-related quality of life as measured by the Duke Health Profile, and Patient Health Questionnaire-9 (PHQ-9). The team used Duke Health Profile results to evaluate the relationship between HrQOL and chronic disease (31). Other services include education, health literacy, preventive medicine, diabetes education, nutrition, exercise, and counseling. The interprofessional team works with existing resources and services to augment care, provide work-force development, and develop a multi-system healthcare service.

### Analysis

This Community Case study is further analysis of our previous report. The report is a convenience sample of patients seen during 2016–2018, using anonymized linked healthcare data from 1400 patients treated on a mobile medical van. To preclude biased estimation of effects when the level of missing data is greater than 5 percent (up to 20% in our sample), we used a missing data imputation approach that uses a “random forest” algorithm implemented in R (Version 3.2.3) in the MissForest package to account for missing data. Consequent to imputation using MissForest, we arrived at a final data set of 894 observations for all variables of interest. We analyzed the 894 charts for baseline prevalence, associations, and contributing factors and measured seven health-related variables (obesity, diabetes, hypertension, high triglycerides, low HDL (high-density lipoprotein), high LDL (low-density lipoprotein), and high total cholesterol) routinely measured on patients in our clinics. We also collected information from two survey instruments routinely administered by clinicians staffing the Mobile Unit. The Duke Health Profile is a 17-item self-report questionnaire measures health-related quality-of-life (HrQoL) (reliability 0.30–0.78) (33) and includes six health measures (physical, mental, social, general, perceived health, and self-esteem) and four dysfunction measures (anxiety, depression, pain, and disability). The Patient Health Questionnaire (PHQ-9) (reliability 0.89 sensitivity of 88% and a specificity of 88% for major depression) is a nine-item instrument that measures depression (34).

Logistic regression analysis and factor component analysis were used to determine potential associations between clinical variables and candidate predictor variables and seven physiological health variables and two survey instruments: The PHQ-9 surveys, used to monitor depression, and the Duke Health-Related-Quality-of-Life survey consisting of 11 domains, were analyzed. The FI was computed over the seven physiological variables, PHQ-9 score, and the 11 domain-specific Duke scores for a total of 19 possible variables.

We used the seven physiological health variables, 17 item scores of the Duke Health Profile, and the PHQ-9 score to calculate the FI. Regressions of the Frailty Index against age were performed in subsamples of males, females, Cameron Park patients, and Indian Hills patients. We assessed the difference between males and females and between Colonias, using the difference between slope and intercept tests (35).

Using a complementary multivariate test, we examined the difference between the vectors of means for all the variables considered by way of Hotelling's T^2^ given as the squared difference of mean vectors scaled against the sample covariance matrix while taking sample size differences into account. Hotelling's T^2^ was computed and then transformed into an F-statistic for statistical inference in r (Version 3.2.3).

## Results

**Figure 1:**
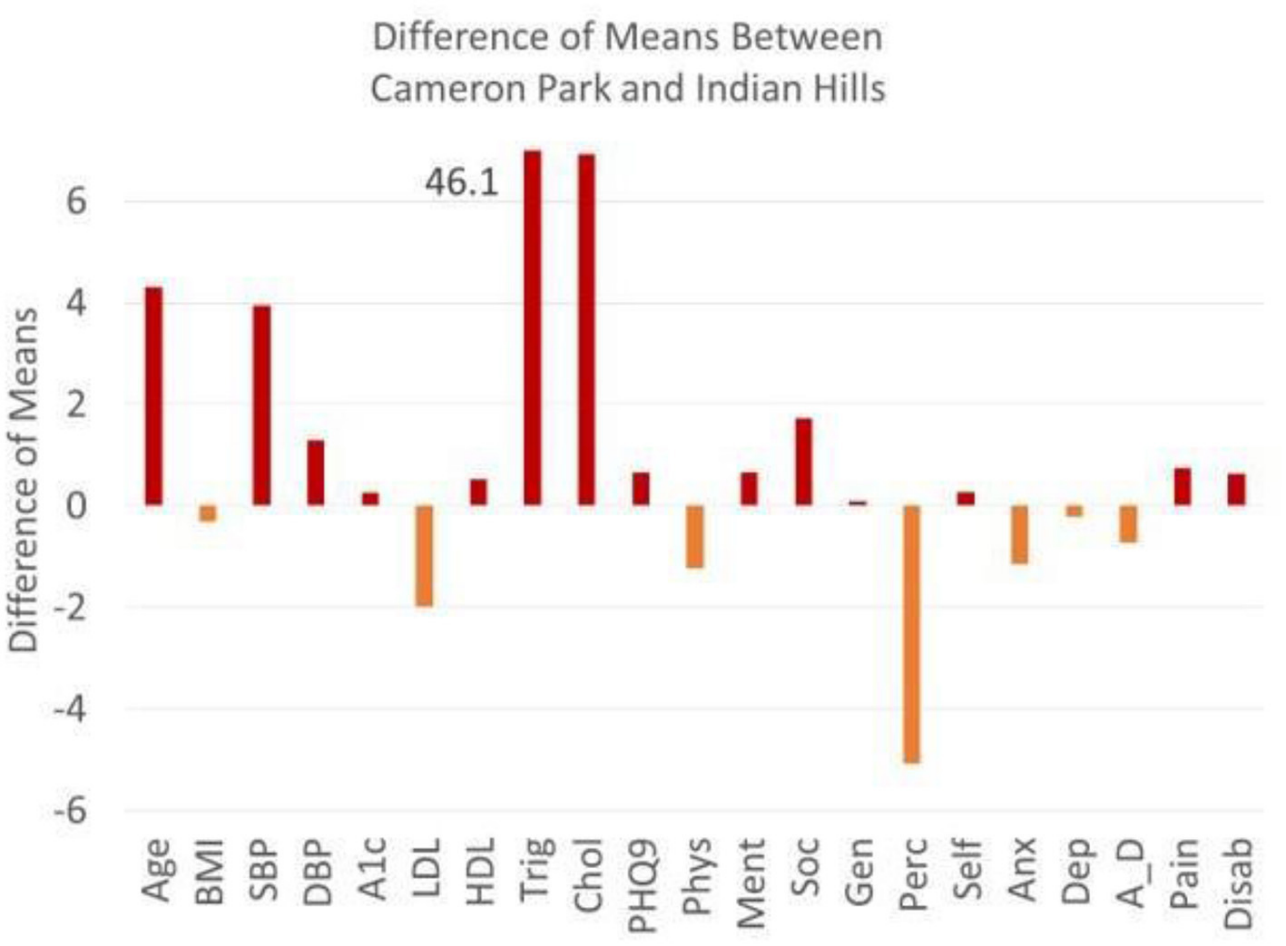
Differences between means between cameron park and Indian hills. The plot of the difference of means as calculated by hoteling T2 is the squared difference of mean vectors scaled against the co-variance matrix for age (yrs), BMI, SBP (systolic blood pressure), DBP diastolic blood pressure), A1c (HbA1C), LDL, HDL, Trig (Triglycerides), Chol (Cholesterol), PHQ9, physical health, mental health, social health, general health, perceived health, self-esteem, anxiety, depression, anxiety-depression, pain, disability. Above zero reflects a higher Cameron Park (red) and below zero reflects a higher Indian Hills Value (orange). Triglyceride means do not fit on the graph and are defined by the means across Colonia.

**Figure 2:**
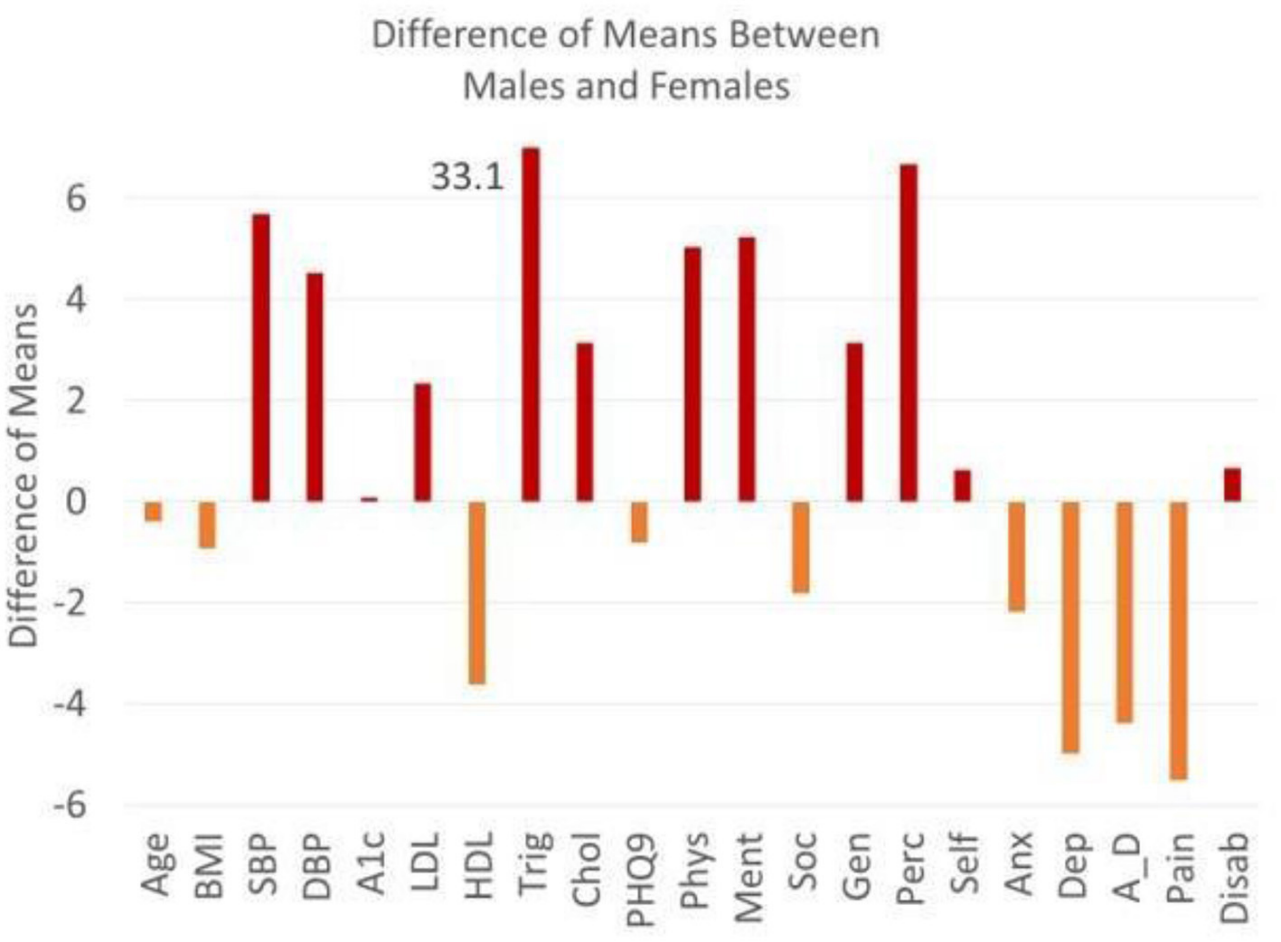
Differences of means between males and females. The plot of the difference of means as calculated by hoteling T2 is the squared difference of mean vectors scaled against the co-variance matrix for age (yrs), BMI, SBP (systolic blood pressure), DBP diastolic blood pressure, A1c (HbA1C), LDL, HDL, Trig (Triglycerides), Chol (Cholesterol), PHQ9, physical health, mental health, social health, general health, perceived health, self-esteem, anxiety, depression, anxiety-depression, pain, disability. Above zero reflects a higher male value (red) and below zero reflects a higher female value (orange). Triglyceride means do not fit on the graph and are defined by the means across gender.

Tables 1, 2 list the variables used to calculate the Frailty Index with corresponding prevalence, standard deviation, and *p* values. Obesity, diabetes, hypertension, and depression are highly prevalent in both Colonias. Men score significantly higher than women in hypertension and hypertriglyceridemia. Men score better than women in all four domains of HrQoL function (physical health, mental health, social health, general health, self-perceived health, and self-esteem). Women score worse in anxiety, depression, anxiety-depression, and pain. The plot of the difference of means as calculated by Hoteling T2 is the squared difference of mean vectors scaled against the covariance matrix for age (in years), systolic blood pressure (SBP), HbA1c (A1c), high-density lipoprotein (HDL), total cholesterol (Chol), physical health (Phys), mental health, perceived health (Perc), anxiety (Anx), anxiety-depression (A_D), pain (pain), and disability (Disab) compared across both Colonias. If we look across Colonias (Figure 2), residents of Cameron Park are older (*P* < 0.001), with higher SBP, HbA1C, low HDL, and high cholesterol measurements. Residents of the two Colonias score similarly between 10 of the 11 domains of the Duke Profile other than perceived health (Figure 1). Males score worse on five of the six domains that measure function (i.e., physical health, mental health, social health, general health, perceived health). For the five domains assessed by the Duke Profile (anxiety, depression, anxiety-depression, pain, and disability), women consistently scored worse than men (Figure 2) in all domains except for the disability domain.

**Table 1:** Total and sex-specific prevalence statistics for clinical outcomes.

**Trait**	**Males (** ***N*** **= 272)**		**Females (** ***N*** **= 622)**		*p*-value^*^
	**Prevalence**	**S.D**.	**Prevalence**	**S.D**.	
Norm Wt.	17	0.02	14	0.01	0.161
Over. Wt.BMI 26–29	31	0.03	29	0.02	0.335
ObeseBMI > 30	53	0.03	57	0.02	0.156
Norm.HbA1c <5.5	39	0.03	37	0.02	0.280
Pre-DMHbA1C 5.5–6.5	29	0.03	31	0.02	0.280
DMHbA1C >6.5	32	0.03	33	0.02	0.488
HTN >140/90	46	0.03	36	0.02	0.002
Cholesterol >200	7	0.02	6	0.01	0.187
Triglycerides	60	0.03	48	0.02	0.000
>200 mg/DLLow HDL-C<40 mg/DL	7	0.02	6	0.01	0.187
Depression	33	0.03	20	0.02	<0.000
PHQ9 >10	17	0.02	19	0.02	0.232

* *Prevalence differences were tested using a difference of proportion Z-statistic that is normally distributed for a one-tailed test. SD, Standard Deviation*.

**Table 2:** Duke health profile by total and sex-specific samples.

**Domain**	**Males**			**Females**			*p*-value^*^
	***N***	**Mean**	**S.D**.	***N***	**Mean**	**S.D**.	
Physical health	272	65.89	22.63	622	60.93	21.55	0.01
Mental health	272	77	19.24	622	71.72	17.62	0.00
Social health	272	66.85	17.03	622	68.8	17.42	0.06
General health	272	70.11	15.6	622	67.04	14.26	0.00
Self-perceived health	272	71.81	27.26	622	65.65	28.58	0.00
Self esteem	272	78.86	15.98	622	78.33	16.02	0.33
Anxiety	272	31.58	18.58	622	33.76	16.53	0.04
Depression	272	29.75	20.98	622	34.76	18.98	0.00
Anxiety-depression	272	26.83	19.19	622	31.23	17.15	0.00
Pain score	272	42.99	28.57	622	48.38	29.94	0.01
Disability	272	8.74	21.29	622	8.03	20.31	0.32

* *Unpaired sample t-test for a one-tailed hypothesis*.

*The first 6 (red) measure function, with high scores indicating greater dysfunction. The second 5 (blue) measure dysfunction, with high scores indicating greater dysfunction*.

**Figure 3:**
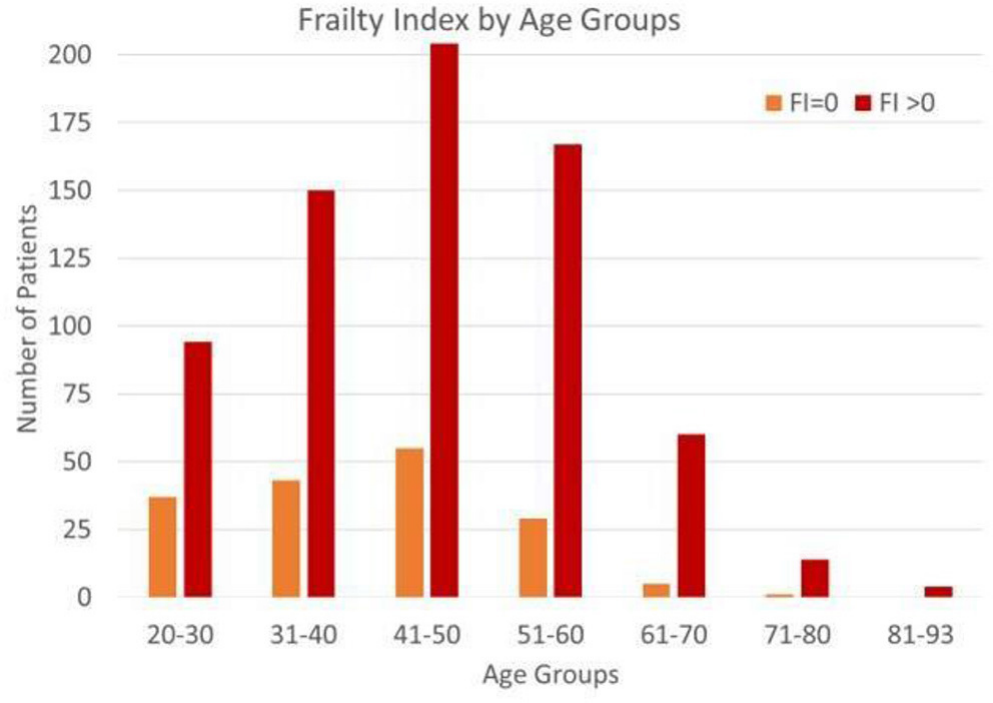
Frailty index by age group. Proportion of FI = 0 and FI greater than zero. The proportion of patients with a non-zero Frailty Index increased significantly (*p* < 0.001) from the younger to other age groups (20–45 vs. 46–93).

Frailty increases with age and peaks between the ages of 40 and 60 years (Figure 3). The proportion of patients with a non-zero Frailty Index increased significantly (*p* < 0.001) from the younger to older age groups (20–45 vs. 46–93). The initial Frailty Index for women is higher than men, but as men age, they decline faster than women (Supplementary Figure 1). The mean Frailty Index for women is higher than that for men (*p* < 0.01), which is consistent with their significantly different mean vectors as inferred from the Hotelling's T-squared result (*p* < 0.001).

The Frailty Index in Indian Hills remained stable with increasing age (Supplementary Figure 2) when compared to Cameron Park (*p* < 0.001), and the Frailty Index was significantly higher in Cameron Park (*p* < 0.02), consistent with their significantly different mean vector (*p* < 0.001).

## Discussion

We developed a Frailty Index calculated with 19 variables from two surveys (Health-related Quality of Life and Depression) and seven clinical measurements. We chose the seven clinical measurements and the two surveys because they are evidence-based screening metrics routinely used in primary care to evaluate for health and quality of life.

The prevalence of chronic disease in the region is high. The residents from Cameron Park Colonia are older, and hyperlipidemia and hypertension are more prevalent in this population than in Indian Hills Colonia. The prevalence of diabetes and obesity (high) are similar in both Colonias. A cross-sectional representation of residents of Indian Hills demonstrated little change of Frailty with age, whereas Frailty increased dramatically with age in the more established Cameron Park, with the peak frailty index scores occurring between 40 and 60 years of age.

Cameron Park is located on the US-Mexico border and is within the boundaries of the city of Brownsville. The proximity of Cameron Park to the border means that many residents can find affordable healthcare a few minutes away in Mexico. Cameron Park appears more stable, and although poor, most residents live in constructed houses rather than manufactured homes, the roads are paved, and city services are available. Housing options, community services, and life in Indian Hills are more problematic. Indian Hills only recently paved roads, and most residents live in trailers and make-shift housing without central air conditioning.

The Frailty Index was calculated against age, and Colonia was regressed against age. Indian Hills had a significantly higher starting frailty, while Cameron Park had a significantly greater change rate with age.

### Implications for Practice

Prior researchers note positive contributors to Frailty in Hispanic dense, cultural and language congruent neighborhoods, suggesting that social capital, positive cultural protection, reduced social vulnerability, and proximity to appropriate language- and culturally based assistance protect against Frailty. The immigrant paradox may also explain the improved health of families in Indian Hills; families residing in Indian Hills may be more willing to seek medical care, support the elderly, or are newer immigrants (13–16). We propose living in a Colonia located within a city exposes residents to worse food choices, social isolation, social vulnerability, and housing insecurity. The rate of decline in Frailty in Cameron Park may be related to social factors, age, job types, immigration status, and increased availability of high caloric foods. Residents of Indian Hills may retain a more traditional diet than Cameron Park, which has ready access to fast food, junk food, and sugary drinks. Perhaps living day-to-day forces residents in Indian Hills to ration their money, influencing their diet, health, and psychosocial well-being.

We also found that women score higher in Frailty than men when young, but the FI for men worsened rapidly with advancing age. Prior studies have attempted to explain gender-based differences found in Frailty (16, 36–38). In our sample, women and men have the same prevalence of disease traits (except for triglyceride levels), yet the frailty indices are higher for women. Socio-behavioral explanations include cultural differences why men may not seek care or accept disability or disease. Hispanic men in the Colonias are involved in more physical labor, their diet is calorically high, and aside from work, they often do not participate in a planned exercise program. Women may suffer from more adverse psychosocial stressors at a young age (as seen by the Duke Profile's five domains that measure dysfunction). Teen pregnancy, large family sizes, intimate partner violence, and women's role in the family structure may explain Frailty's variance in younger women (28).

The Frailty Index can be used to identify at-risk patients that live in vulnerable environments. Those residents who score high in depression or low in domains of quality of life and suffer from obesity, hypertension, and depression may be identified earlier and focused interventions provided at the healthcare and public health level. The Frailty Index is easy to calculate with evidence-based data routinely collected.

### Implications for Research

The findings in this report are subject to several limitations. The sample is a convenience sample, the data are cross-sectional, and the community case study represents a picture of what is going on in the Colonias. The acute decline of Frailty in men could be related to early death, migration away from the community, and decreased healthcare access and there may be other contributors (social, cultural, and biological) that influence decline to frailty. Finally, although evidence-based metrics, the variables used to calculate may be insufficient to measure Frailty in our population. Evidence-based research is required to determine the significant contributors to Frailty and determine a causal link.

## Conclusion

Patients seeking care through a mobile clinic serving Colonias in South Texas suffer from a high prevalence of diabetes, hypertension, obesity, depression, hypertriglyceridemia, and depression. The Frailty Index is suggested by the US Preventive Services Task Force Recommendations for Primary Care Practice as a measure and is a good predictor of Frailty.

Although social determinants of health and health equity are strong predictors of chronic disease, it is essential to study how these stressors affect immigrants living in underserved areas.

Life stressors for vulnerable populations contribute to increased Frailty with age, and the variables are biopsychosocial based. The prevalence of chronic disease and depression in our region is epidemic. Hispanic patients that seek healthcare from a mobile clinic serving Colonias in South Texas suffer from high rates of diabetes, hypertension, obesity, depression, hypertriglyceridemia, and they score poorly on health-related quality of life measures. Research in highly vulnerable populations, such as residents of the Colonias of the US-Mexico border, is inadequate. These preliminary data can serve as background evidence to design future research to incorporate stressors and social support in examining those who become frail, especially in minority populations. Although social determinants of health and health equity are strong predictors of risk for chronic disease, it is crucial to study how these stressors affect minority residents and immigrants living in medically underserved areas. The Frailty Index, however, provides information for the accumulation of deficits and may help clinicians to focus on interventions to prevent eventual Frailty.

## Data Availability Statement

The datasets presented in this study can be found in online repositories. The names of the repository/repositories and accession number(s) can be found below: manusov, eron (2021), “Mobile Medical Van in the South Texas Colonias”, Mendeley Data, V2, 10.17632/zz2b3wdf5r.2.

## Ethics Statement

The studies involving human participants were reviewed and approved by University of Texas Rio Grande Valley Institutional Review Board. The patients/participants provided their written informed consent to participate in this study.

## Author Contributions

EM was the Principal Investigator on the United Health Foundation grant, collected data, analyzed data, wrote, and was substantially involved in editing the manuscript. CG analyzed data, wrote, and was substantially involved in editing the manuscript. VD analyzed data and completed the statistical analysis, was substantially involved in editing. GM-M analyzed data, contributed to the manuscript, and was substantially involved in editing. SW-B analyzed data and was substantially involved in editing. All authors listed have made a substantial, direct and intellectual contribution to the work, and approved it for publication.

## Conflict of Interest

The authors declare that the research was conducted in the absence of any commercial or financial relationships that could be construed as a potential conflict of interest.

## Publisher's Note

All claims expressed in this article are solely those of the authors and do not necessarily represent those of their affiliated organizations, or those of the publisher, the editors and the reviewers. Any product that may be evaluated in this article, or claim that may be made by its manufacturer, is not guaranteed or endorsed by the publisher.

## Abbreviations

FI: Frailty Index
SEL: Socioeconomic Level
HDL: High-Density Lipoprotein
LDL: Low-Density Lipoprotein
PHQ-9: Patient Health Questionnaire 9
HrQOL: Health-Related Quality of Life
SBP: Systolic Blood Pressure
HbA1C: Hemoglobin A1C.
