# Supplementary figures and images for: Frailty Index in the Colonias on the US-Mexico Border: A Special Report
Source: Front Med (Lausanne). 2021 Aug 18;8:650259. doi: 10.3389/fmed.2021.650259 (PMC8416248; doi:10.3389/fmed.2021.650259)

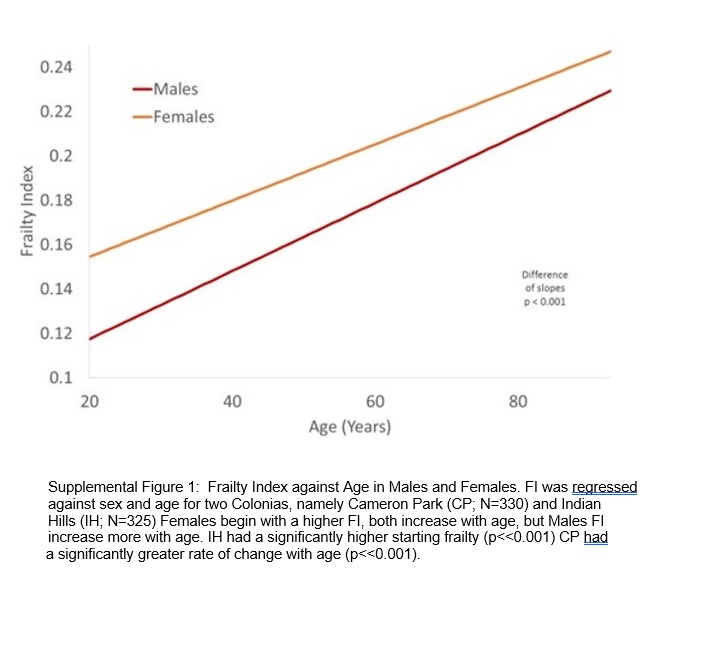

Supplement: Supplementary file 1 [file Image_1.JPEG]

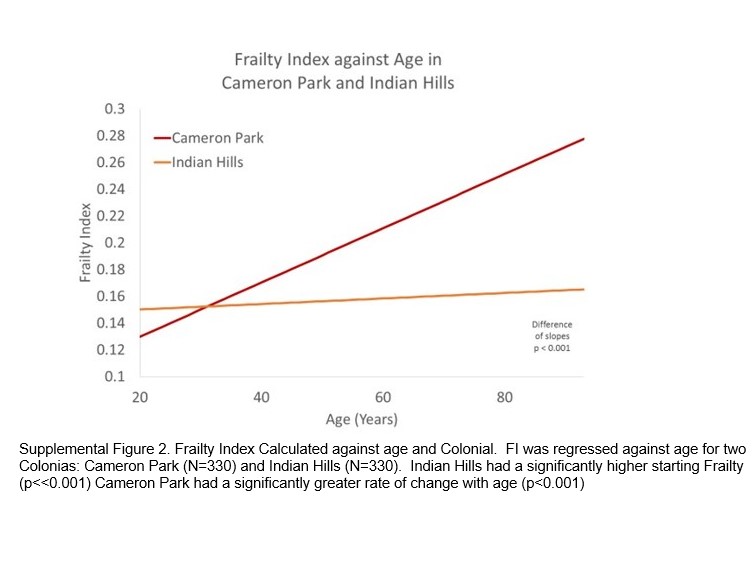

Supplement: Supplementary file 2 [file Image_2.JPEG]
